# Serologic Responses in Healthy Adult with SARS-CoV-2 Reinfection, Hong Kong, August 2020

**DOI:** 10.3201/eid2612.203833

**Published:** 2020-12

**Authors:** Paul K.S. Chan, Grace Lui, Asmaa Hachim, Ronald L.W. Ko, Siaw S. Boon, Timothy Li, Niloufar Kavian, Fion Luk, Zigui Chen, Emily M. Yau, Kin H. Chan, Chi-hang Tsang, Samuel M.S. Cheng, Daniel K.W. Chu, Ranawaka A.P.M. Perera, Wendy C.S. Ho, Apple C.M. Yeung, Chit Chow, Leo L.M. Poon, Sophie A. Valkenburg, David S.C. Hui, Malik Peiris

**Affiliations:** The Chinese University of Hong Kong, Hong Kong, China (P.K.S. Chan, G. Lui, S.S. Boon, T. Li, F. Luk, Z. Chen, W.C.S. Ho, A.C.M. Yeung, C. Chow, D.S.C. Hui);; The University of Hong Kong, Hong Kong (A. Hachim, R.L.W. Ko, N. Kavian, E.M. Yau, K.H. Chan, C. Tsang, S.M.S. Cheng, D.K.W. Chu, R.A.P.M. Perera, L.L.M. Poon, S.A. Valkenburg, M. Peiris)

**Keywords:** coronaviruses, COVID-19, SARS-CoV-2, reinfection, serology, antibodies, immune response, coronavirus disease, respiratory infections, severe acute respiratory syndrome coronavirus 2, 2019 novel coronavirus disease, zoonoses, viruses, Hong Kong

## Abstract

In March 2020, mild signs and symptoms of coronavirus disease developed in a healthy 33-year-old man in Hong Kong. His first infection did not produce virus neutralizing antibodies. In August, he had asymptomatic reinfection, suggesting that persons without a robust neutralizing antibody response might be at risk for reinfection.

Severe acute respiratory syndrome coronavirus 2 (SARS-CoV-2) is the causative agent of coronavirus disease, which has caused a pandemic in humans. Whether SARS-CoV-2 infection induces serologic immunity and the duration of that immunity is unknown. In humans, reinfection with seasonal coronaviruses occurs naturally and in experimental conditions (*1*,*2*).

Within 30 days after infection, most persons with SARS-CoV-2 begin producing antibodies against the spike and N proteins of the virus (*3*,*4*). An outbreak of SARS-CoV-2 on a fishing vessel showed that persons with prior neutralizing antibodies against SARS-CoV-2 were not reinfected (*5*). We analyzed the serologic and cytokine responses of a patient who had 2 episodes of SARS-CoV-2 infection (*6*). These findings have implications for population immunity generated from natural infection or vaccines.

On March 23, 2020, fever, headache, cough, and sore throat developed in a 33-year-old Caucasian man with no underlying conditions in Hong Kong. Six days later, the patient was admitted to the hospital with mildly elevated levels of alanine aminotransferase (73 U/L, reference range <50 U/L) and lactate dehydrogenase (236 U/L, reference range 106–218 U/L). Chest radiographs did not show any infiltrates. He tested negative for hepatitis B surface antigen and antibodies against HIV and hepatitis C virus. He had IgG against measles virus and varicella zoster virus. Symptoms resolved completely within 3 days. A sample of the patient’s deep throat saliva tested positive for SARS-CoV-2 RNA by reverse transcription PCR (RT-PCR). During days 6–20 after symptom onset, the patient tested positive 7 more times; RT-PCR cycle thresholds ranged from 31 through 36 (Figure). He was isolated in the hospital until twice testing negative for SARS-CoV-2 by RT-PCR, on days 21 and 22. At a follow-up visit on day 43 (i.e., May 5, 2020), he was asymptomatic and had resumed his usual work. We took serum samples on days 10 and 43 (Figure).

**Figure:**
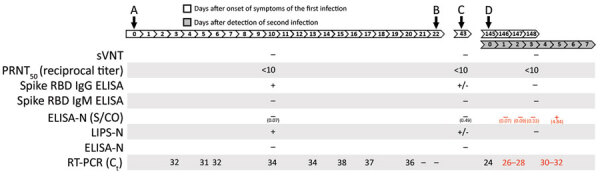
Timeline of primary infection and reinfection with severe acute respiratory syndrome coronavirus 2, Hong Kong, August, 2020. A) Onset. B) Discharge. C) Clinical follow-up. D) Mandatory testing. Black font indicates data from this investigation; red font indicates data from To et al. (*6*). C_t_, cycle threshold; ELISA-N, enzyme linked immunosorbent assay for N protein; LIPS, luciferase immune precipitation assay; PRNT_50_, 50% plaque reduction neutralization test titer; RBD, receptor binding domain; RT-PCR, reverse transcription PCR; S/CO, ratio of optical density readings of sample divided by cutoff (ratio of >1.4 considered positive); sVNT, surrogate virus neutralization test; +, positive; –, negative; +/–, borderline.

On August 15, 2020, the patient returned to Hong Kong after a 1-week trip in Spain. As a part of border surveillance, he submitted a deep throat saliva sample for RT-PCR; this sample tested positive for SARS-CoV-2 RNA. He remained asymptomatic throughout his second infection. The clinical course of this second episode has been reported elsewhere (Figure) (*6*). We confirmed the previous report (*6*) that viruses from the first and second infection of this patient were phylogenetically distinct (Appendix Figure 1), demonstrating reinfection. We collected baseline serum on day 3 after detection of reinfection (day 148 after symptom onset of his first infection) to infer his probable preinfection serologic results.

The 50% plaque reduction neutralization test (*3*) and surrogate virus neutralization test (*7*) on the serum samples collected on days 10, 43, and 148 did not detect antibodies against SARS-CoV-2. ELISA showed decreasing titers of serum IgG against the spike receptor-binding domain (RBD) of SARS-CoV-2; on day 148, the patient tested negative for these antibodies (*3*). All 3 serum samples tested negative for IgM against spike RBD (Appendix Figure 2). On day 10, the patient tested negative for N-specific serum IgG by chemiluminescent microparticle immunoassay assay (Abbott, https://www.corelaboratory.abbott) and indirect microtiter plate enzyme immunoassay; he tested weakly positive on day 43 in a validated luciferase immunoprecipitation assay (*4*) (Figure). As reported previously (*6*), a strong antibody response to N protein developed by day 5 of reinfection. This response suggests that antibody against SARS-CoV-2 developed on reinfection.

Levels of adaptive cytokine interleukin-2 were elevated on days 10 and 43 (Appendix Figure 3, panels A, B). Reinfection coincided with a stronger interleukin-21 memory type response on day 148 than on days 10 and 43.

Previous studies show that most patients with mild, severe, or asymptomatic SARS-CoV-2 infection produce neutralizing antibodies and antibodies against spike RBD and N proteins (*3*,*4*). This case was unusual because the patient had low or undetectable levels of neutralizing and binding antibodies against multiple viral proteins during his primary infection and acute stage of asymptomatic reinfection. He was not immunodeficient because he had IgG against measles and varicella zoster viruses and no history of recurrent infections. The virus from the first infection had a truncation in the 58AA open reading frame 8 gene, which mediates immune evasion through downregulation of major histocompatibility complex and interferon responses (Y. Zhang et al., unpub. data, https://www.biorxiv.org/content/10.1101/2020.05.24.111823v1) (*8*). However, it is unclear if this mutation contributed to the patient’s lack of antibody production.

Reasons for this patient’s unusual response need to be further investigated. He recovered from his primary infection within 3 weeks, and his secondary infection was asymptomatic. These findings indicate that, in the absence of primary neutralizing antibodies, T cells and mucosal immunity might have played a critical role in resolving the infection. Given the unusual antibody response in this patient to his first infection, researchers must be cautious about generalizing more widely from this patient’s experience.

Appendix Additional information for serologic responses in SARS-CoV-2 reinfection, Hong Kong, 2020.
