# Supplementary material for: Serologic Responses in Healthy Adult with SARS-CoV-2 Reinfection, Hong Kong, August 2020
Source: Emerg Infect Dis. 2020 Dec;26(12):3076–8. doi: 10.3201/eid2612.203833 (PMC7706979; doi:10.3201/eid2612.203833)
Supplement: Appendix — Additional information for serologic responses in SARS-CoV-2 reinfection, Hong Kong, 2020. [file 20-3833-Techapp-s1.pdf]

Article DOI: https://doi.org/10.3201/eid2612.203833

Serologic Responses in Healthy Adult with SARS-CoV-2 Reinfection, Hong Kong, August, 2020

Appendix

Methods

Patient Follow-Up

Written, informed consent was obtained from the patient to participate in our study on COVID-19, which was approved by The Joint Chinese University of Hong Kong-New Territories East Cluster Research Ethics Committee.

Plaque Reduction Neutralization Test (PRNT)

PRNT antibodies were detected as previously described (*1*). Briefly, serial dilutions of each serum sample were incubated with 30–40 plaque-forming units of viruses for 1 h at 37°C. The virus–serum mixtures were added onto pre-formed Vero E6 cell monolayers and incubated for 1 h at 37°C in 5% CO_2 incubator. The cell monolayer was then overlaid with 1% agarose in cell culture medium. After 3 d of incubation, the plates were fixed and stained. Antibody titers were defined as the highest serum dilution that resulted in $\geq$50% ($PRNT_{50}$) reduction in the number of virus plaques.

Surrogate Virus Neutralization Assay

Neutralizing antibodies were also tested by the SARS-CoV-2 surrogate virus neutralization test kits (GeneScript USA, Inc, New Jersey) according to the manufacturer's instructions (*2*). Briefly, the test sera (60 µL), the positive and negative controls were diluted at 1:10 and mixed with an equal volume of horseradish peroxidase (HRP) conjugated SARS-CoV-2 spike receptor binding domain (RBD) protein and incubated for 30 min at 37°C. Then, 100 µL of each mix was added to the wells on the microtiter plate coated with ACE-2 receptor, the plate was sealed and incubated at 37°C for 15 min. The plates were then washed with wash-solution,

tapped dry and 100 μL of 3,3′,5,5′-Tetramethylbenzidine (TMB) solution was added to each well and incubated in the dark at room temperature for 15 min. The reaction was stopped by addition of 50 μL of Stop Solution to each well and the absorbance read at 450 nm in an ELISA reader. Assuming the positive and negative controls gave the recommended OD450 values, the % inhibition of each serum was calculated as (1 – OD value of the sample/OD value of the negative control) × 100. An inhibition of $\geq$20% is regarded as a positive result while that <20% is negative (*2*).

Luciferase Immunoprecipitation (LIPS) Assay

The LIPS assay was initially described by Burbelo et al. (*3*) adapted to SARS-CoV-2 by us as previously described (4). Briefly, Renilla luciferase tagged SARS-CoV-2 N antigens were produced from COS1 cells, and antigen equalized to 10^7 luciferase units for each serological test. Serum (heat inactivated and diluted 1:100 in Buffer A (50 mM Tris, pH 7.5, 100 mM NaCl, 5 mM MgCl2, 1% Triton X-100)) was incubated with 10^7 LU of (Ruc)-N antigen for 1 h with shaking at 800 rpm. Ultralink protein A/G beads were added to the (Ruc)-antigen and serum mixture in a 96-deep-well polypropylene microtiter plate and incubated for 1 h with shaking at 800 rpm. The entire volume was then transferred into HTS plates and washed 10 times with Buffer A, and then twice with PBS. The plate was read using QUANTI-Luc Gold substrate (Invivogen, USA) as per manufacturer's instructions on a Wallac MicroBeta JET luminometer 1450 LSC & Luminescence counter and its software for analysis (PerkinElmer, USA). Experimental controls include no-serum blank wells with (Ruc)-antigens and negative control serum from age-matched noninfected patient plasma collected prior to the COVID-19 pandemic (n = 20). The background corresponds to the LU signal from each Ruc-fusion antigen with protein A/G and substrate with no serum. The cutoff limits were derived from the mean value plus 3 × SD of the negative controls.

Virus Full-Genome Sequencing

We used the methods previously described by us (*5*). Briefly, virus genome was reverse transcribed with multiple gene-specific primers targeting different regions of the viral genome. The synthesized cDNA was then subjected to multiple overlapping 2-kb PCRs for full-genome amplification. PCR amplicons obtained from the same specimen were pooled and sequenced using MiSeq sequencing platform (Illumina). Sequencing library was prepared by Nextera XT

DNA library prep Kit (Illumina) following standard protocol. Generated sequencing reads were mapped to a reference virus genome by BWA (*6*), and genome consensus was generated by Geneious version 11.1.4 (https://www.geneious.com).

Anti-SARS-CoV-2 N IgG by In-House Indirect Microtiter Plate ELISA

Purified glutathione S transferase (GST)-tagged full-length nucleocapsid fusion protein (GST-N) of SARS-CoV-2 (1 μg/mL) was coated onto Immulon 2 HB 96-well microtiter plate (ImmunoChemistry, USA) in bicarbonate buffer (pH 9.6) overnight at 4°C. Blocking buffer containing 0.25% BSA (Sigma, USA) in 1 × TBS was added into wells, followed by adding 100 μL of human plasma or serum diluted at 1:100 in 1 × TBS containing 0.2% Tween-20 at 37°C for 1 h. The plates were washed and incubated with horse-peroxidase-labelled goat antihuman IgG (Invitrogen, USA) at room temperature for 30 min. After washing, detection was carried out by adding 3,3′,5,5′-Tetramethylbenzidine (TMB) substrate (Sigma, USA) in dark and the reaction was stopped by adding 2 M of sulfuric acid. The results were read at 450 nm using a VICTOR 3 Multilabel Plate Reader (PerkinElmer, USA). Mean OD of negative controls plus 3 standard deviations was defined as cut-off for reactive samples. Validation using 50 convalescent samples of patients with confirmed SARS-CoV-2 infection collected at ≥30 days from illness onset revealed 100% sensitivity, and from 50 samples taken from healthy persons before November 2019 revealed 100% specificity.

IgG Against Varicella Zoster Virus and Measles by Commercial Microtiter Plate ELISA

Commercial assays for measles IgG and varicella zoster virus IgG (Trinity Biotech, USA) were used according to the manufacturer's instructions. OD index of ≥1.1 is regarded as positive in both assays. The serum sample collected at Day-10 from this patient was positive for varicella zoster virus IgG (OD: 6.358) and measles IgG (OD: 2.553).

Cytokine Bead Array

A custom 10 cytokines LegendPlex (Biolegend, USA) panel which included CCL3 (MIP-1α), IFN-α2, IFN-γ, IL-2, IL-4, IL-5, IL-9, IL-10, IL-13, and IL-21 was used according to the manufactures instructions. Samples were acquired by flow cytometry on a FACS Attune (Invitrogen) and analyzed with LegendPlex software as per manufacturer's instructions. Samples included case 564 (day 10, 43 and 148, and negative controls (healthy donors, pre-pandemic, no current infection, n=7). Patient sera and controls were run in parallel on the same occasion.

References

1. Perera RA, Mok CK, Tsang OT, Lv H, Ko RL, Wu NC, et al. Serological assays for severe acute respiratory syndrome coronavirus 2 (SARS-CoV-2), March 2020. Euro Surveill. 2020;25:2000421. PubMed https://doi.org/10.2807/1560-7917.ES.2020.25.16.2000421

2. Tan CW, Chia WN, Qin X, Liu P, Chen MI, Tiu C, et al. A SARS-CoV-2 surrogate virus neutralization test based on antibody-mediated blockage of ACE2-spike protein-protein interaction. Nat Biotechnol. 2020;38:1073–8. PubMed https://doi.org/10.1038/s41587-020-0631-z

3. Burbelo PD, Ching KH, Klimavicz CM, Iadarola MJ. Antibody profiling by Luciferase Immunoprecipitation Systems (LIPS). J Vis Exp. 2009; 32:1549. PubMed https://doi.org/10.3791/1549

4. Hachim A, Kavian N, Cohen CA, Chin AWH, Chu DKW, Mok CKP, et al. ORF8 and ORF3b antibodies are accurate serological markers of early and late SARS-CoV-2 infection. [Erratum in: Nat Immunol. 2020 Aug 17; Epub ahead of print.] Nat Immunol. 2020 Aug 17 [Epub ahead of print]. PubMed

5. Sit THC, Brackman CJ, Ip SM, Tam KWS, Law PYT, To EMW, et al. Infection of dogs with SARS-CoV-2. Nature. 2020 May 14 [Epub ahead of print]. https://doi.org/10.1038/s41586-020-2334-5 PMID: 32408337

6. Li H, Durbin R. Fast and accurate short read alignment with Burrows-Wheeler transform. Bioinformatics. 2009;25:1754–60. PubMed https://doi.org/10.1093/bioinformatics/btp324

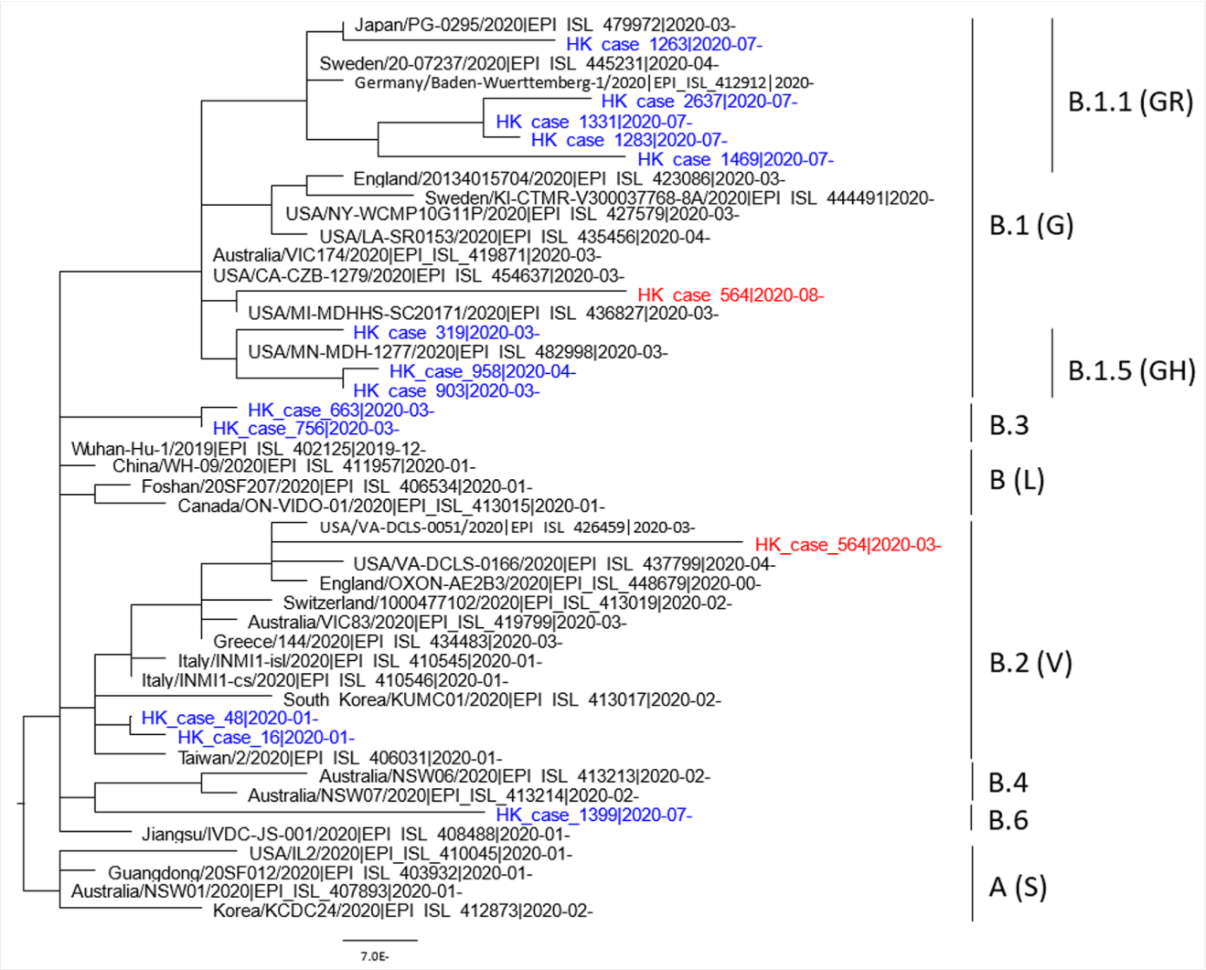

Appendix Figure 1. Phylogenetic tree of selected severe acute respiratory syndrome coronavirus 2 isolates. Red indicates viruses isolated from patient 564, Hong Kong, 2020. Patient 564 was sampled during his first infection in March and the second infection in August. Blue indicates viruses from other recent Hong Kong cases. Phylogenetic tree constructed using PhyML. Symptom onset date (YYYY-MM-DD) and GISAID access no. of the reference genomes are shown in virus taxa. Virus lineages are indicated.

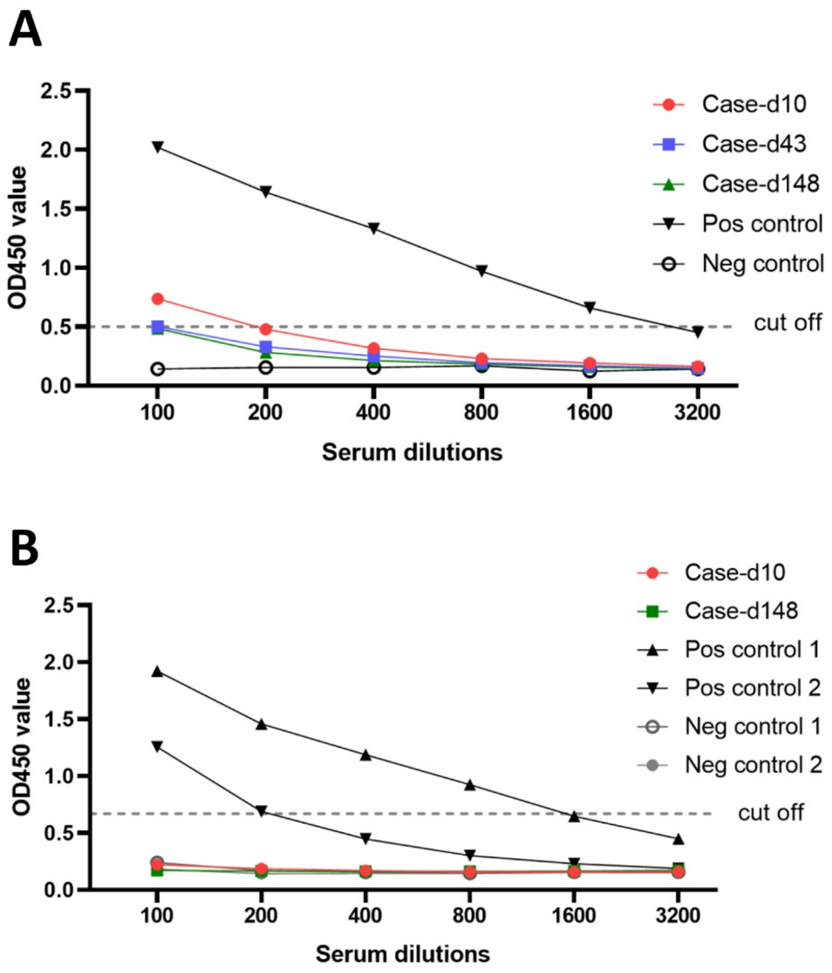

Appendix Figure 2. Serum IgG and IgM responses to spike receptor binding domain in patient with selected severe acute respiratory syndrome coronavirus 2 reinfection, Hong Kong, 2020. Serial dilutions of serum samples from the patient were tested in ELISA selective for IgG (A) and IgM (B) against SARS-CoV-2 receptor binding domain. Positive and negative control serum samples included for comparison. The dotted line represents the cut-off in the previously validated assay (*1*).

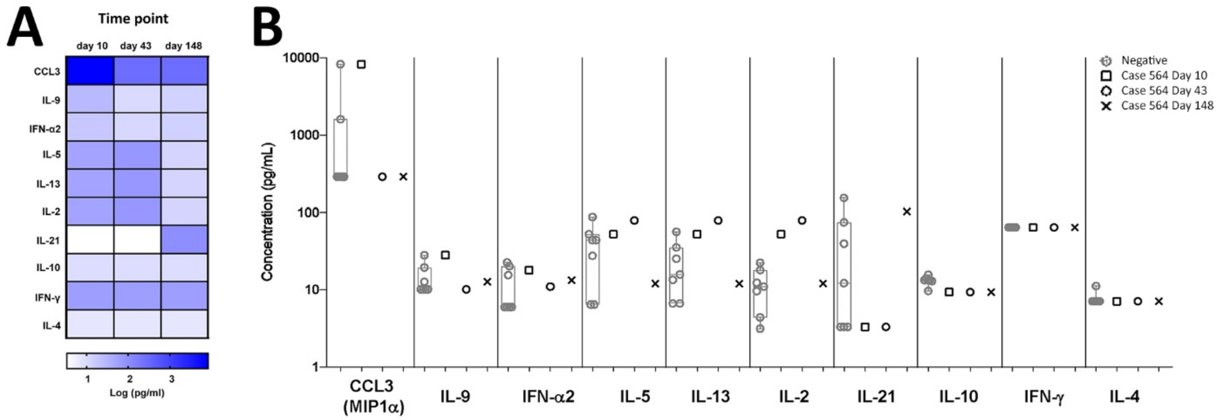

Appendix Figure 3. Pro- and antiinflammatory cytokines and chemokines measured by cytokine bead array in a patient with severe acute respiratory syndrome coronavirus 2 reinfection, Hong Kong, 2020. (A) Cytokine and chemokine levels in patient on days 10, 43, and 148 after symptom onset. (B) The patient's response at different time points (black symbols) were compared to those of 7 negative controls (open gray circles). Data represent individual responses. Box plots show interquartile ranges. IFN, interferon; IL, interleukin; CCL3, chemokine (C-C motif) ligand 3; MIP-1α, macrophage inflammatory protein-1 α.
